# Brazilian Amazonian microorganisms: A sustainable alternative for plant development

**DOI:** 10.3934/microbiol.2025008

**Published:** 2025-02-07

**Authors:** Monyck Jeane dos Santos Lopes, Aline Figueiredo Cardoso, Moacyr Bernardino Dias-Filho, Ely Simone Cajueiro Gurgel, Gisele Barata da Silva

**Affiliations:** 1 Coordination of Botany, Biotechnology Laboratory of Propagules and Seedlings, Museu Paraense Emílio Goeldi, Pará, Brazil; 2 Instituto Tecnológico Vale, Brazil; 3 Brazilian Agricultural Research Corporation (Embrapa), Pará, Brazil; 4 Federal Rural University of Amazonia (UFRA), Belém, PA, Brazil

**Keywords:** Amazon biome, inoculants, plant growth-promoting bacteria, sustainability

## Abstract

Plant growth-promoting microorganisms (PGPM) are a sustainable and promising alternative to enhance agricultural production. The Brazilian Amazon, and its mostly unexplored biodiversity, have great potential for identifying and isolating beneficial microorganisms to develop sustainable protocols for plant production with less environmental damage and to meet the increasing demand for food production. Thus, this study aimed to synthesize the findings over the last decade on microorganisms from the Amazon biome that promote plant growth while also addressing the challenges and prospects of this biotechnology. Rhizobacteria (PGPR) and fungi native to the Amazon have been shown to enhance the development of various crops, spanning agriculture and forestry, including palm cultivation and forage crops. The potential of PGPM in the Brazilian Amazon discussed throughout this review highlights the importance of further research in this region. Amazon PGPM is promising for use in the inoculant industry, which would contribute to the agricultural production of diverse crops, reduce costs, minimize the use of chemical inputs, mitigate adverse environmental impacts, and support the conservation of the Amazon biome. Furthermore, the advancement of knowledge in this region holds a great potential, thus offering access to different strains for the formulation of new inoculants that can, in a more sustainable manner, enhance the productivity of various crops, thereby promoting global food security.

## 1. Introduction

The increase in the world population, which is more than 8 billion, raises concerns about food security and will require an increased food production. With projections indicating a population of 9.7 billion by 2050, it is necessary to increase food production by 70%. However, this expansion occurs in the context of limited natural resources and an increasing dependence on chemical inputs, such as fertilizers, whose intensified use has severe environmental implications. To address this demand, agricultural production must be sustainably optimized within areas currently under production without a further expansion of agricultural lands, thereby preventing deforestation and reducing negative environmental impacts [1],[2].

To support sustainable development, in 2015, the United Nations (UN) launched the 2030 agenda, with 17 goals to be achieved, aiming to end poverty, protect the environment, and guarantee peace and prosperity [3]. However, the challenges in achieving these objectives have increased due to climate change, the COVID-19 pandemic, and geopolitical conflicts such as the war between Russia and Ukraine [4]. Additionally, this conflict raises concerns about the food demand because Russia and Ukraine are important producers of essential cereals for human consumption, such as wheat, corn, and barley. Moreover, Russia is a large exporter of fertilizers [5].

Among several objectives, the 2030 Agenda proposes solutions to hunger, climate change, and inequality, and aims to mitigate the risks associated with food security [4]. In this context, the use of beneficial microorganisms for plant development stands out as a sustainable and promising alternative.

Microbial technology is one of the most promising alternatives to improve agricultural production, with a decreased use of fertilizers and other chemical inputs [6]. This technology has the potential to mitigate harmful effects on vegetation caused by climate change, increase the quality of agricultural products, and control plant pathologies and pests, thus contributing to sustainable development, environmental preservation, and food security [7]–[9]. Plant growth-promoting microorganisms (PGPM), such as rhizobacteria (PRPR) and fungi, have been shown to act as biostimulants and biological control agents, enhance plant development and health, increase agricultural productivity, strengthen plant resistance to adverse conditions, and reduce the use of chemical inputs [7],[8].

New microorganisms capable of sustainably increasing the crop productivity and promoting food security have the potential to be found in the biome Amazon, as this region has a significant biodiversity, with 60% of the region in the Brazilian territory [10]. Brazil is among the world's largest agricultural producers, thus playing a pivotal role in ensuring global food security. However, with a growing population and increasing demands for food production, the country faces significant challenges, including soil degradation, an overreliance on chemical inputs, and an urgent need for more sustainable practices. These challenges are further exacerbated by climate change, which introduces additional stressors such as drought and extreme weather events. Despite this, the vast biodiversity of the Brazilian Amazon remains largely unknown and underexplored [11],[12]. In this context, adopting innovative and environmentally friendly solutions becomes essential. The use of PGPM emerges as a promising strategy to enhance the agricultural productivity while minimizing the environmental impacts.

In the last decade, research conducted using microorganisms, with *Burkholderia pyrrocinia Pseudomonas fluorescens*, *Trichoderma asperellum*, *Bacillus* sp., *Acaulospora*, and *Glomus* originating from the rich biodiversity of the Brazilian Amazon has elucidated their benefits for several species of plants, including the association as a potential mechanism for its outstanding ecological success [13]–[16]. Research results such as these need to be better explored to apply them to different plants and crops in the Amazon and other regions. Disseminating research on microbial and plant interactions is essential for the Amazonian region to gain visibility and increase funding for research using microorganisms from the Amazon that are beneficial to plants and those that can help to achieve a balance between the expansion of agricultural production and environmental conservation.

Therefore, considering the potential of the Brazilian Amazon for agricultural production, the objective of our study was to review publications on PGPM found in the Amazon biome, thereby covering research developed during the last decade on microorganisms from this region that have been shown to be beneficial to plant development. Furthermore, we discuss the challenges and perspectives of research with this biotechnology in the Amazon and highlight efforts to develop sustainable solutions for agriculture in the Amazon based on PGPM.

## 2. Materials and methods

We performed a bibliographic survey using the Web of Science, Scopus, Google Scholar, Science Direct, Wiley Online Library, Scielo, and PubMed databases. Original articles published in the last decade in English, Spanish, or Portuguese were included. In agreement with the research objectives, only articles that used Amazonian microorganism species that examined beneficial interactions between these species and plants were included. The terms used for this research, in Portuguese and English, were as follows: “PGPR AND Amazonia”, “PGPR AND Brazil”; fungi, plants AND Amazon; rhizobacteria, plants AND Amazon; microorganisms AND plants Amazon; PGPM AND Amazon AND plant; microorganism AND plant AND amazon; Trichoderma AND plant AND Amazon; rhizobacteria AND fungi AND plants AND Amazon; and microorganism AND plant AND “state of the Brazilian Amazon”.

Original articles were selected which clearly stated that the analyzed microorganisms came from locations in the Brazilian Amazon. The most frequent terms found in the keywords of the selected articles were used to create a visual representation, called a word cloud. In this word cloud, the size of the words is correlated with the abundance of times the term was found in the selected searches. Based on the results of the bibliographic survey, this article was subdivided into the following sections: Amazon plant growth-promoting microorganisms, microorganisms in plant development, and microbial biotechnology in the Brazilian Amazon: challenges and prospects challenges.

## 3. Amazon PGPM

Research on plant growth-promoting microorganisms, including rhizobacteria and fungi, from the Brazilian Amazon has revealed significant benefits for a range of economically and socially importance plant species. These microorganisms enhance plant development, nutrition, and productivity, while also improving the tolerance of plants against abiotic and biotic stresses (Table 1).

Among the Amazonian microorganisms, bacteria such as *Pseudomonas* sp., *Burkholderia* sp., and *Bacillus* sp., as well as fungi like *Trichoderma* sp., stood out the most as beneficial to plants. The most common plants investigated in these studies were oil palm, açaí, and pasture grasses, thus reflecting their economic importance in the Amazon region.

**Table 1.** Original articles published in the last decade on species of microorganisms from the Brazilian Amazon that promoted plant growth.

Microorganism	Benefits	Plant	Brazil State	Reference
*Ramichloridium* sp.	Biological control of anthracnose	*Euterpe* sp. Mart.	Acre	[17]
*Colletotrichum siamense*	Biological control of anthracnose	*Paullinia cupana* Kunth.	Amazonas	[18]
*Trichoderma* sp.	Increase in growth and efficiency in phosphorus absorbing	*Glycine max* (L.) Merr.	Amazonas	[19]
*Burkholderia ambifaria*	Increase in carbohydrate and proline contente	*Paullinia cupana* Kunth.	Amazonas	[20]
*Acaulospora Glomus*	Ecological success in degraded áreas	*Attalea speciosa* Mart. ex Spreng.	Maranhão	[14]
*B. pyrrocinia P. fluorescens T. asperellum*	Anatomical modifications	*Oryza sativa* L.	Pará	[13]
*Pseudomonas fluorescens Burkholderia pyrrocinia*	Modifies anatomy and increases nutrient concentration	*Brachiaria brizantha* syn. *Urochloa brizantha* (Hochst. ex A.Rich.) R.D.Webster	Pará	[15]
*Bacillus strains Rhizophagus intraradices*	Increased soybean	*Glycine max* (L.) Merr.	Pará	[16]
*Burkholderia pyrrocinia*	Increase in biomass, height, ground cover and nutrient	*Zoysia japônica* Steud.	Pará	[21]
*Pseudomonas fluorescens Burkholderia pyrrocinia*	Increases growth and biomass	*Brachiaria brizantha*	Pará	[22]
*Pseudomonas fluorescens Burkholderia pyrrocinia*	Mitigates the shading effects	*Brachiaria brizantha*	Pará	[23]
*Pseudomonas fluorescens*	Modifies leaf anatomy, increases growth and productivity	*Lactuca sativa* L.	Pará	[24]
*Trichoderma harzianum*	Higher germination rates	*Vigna unguiculata* L. Walp	Pará	[25]
*Bacillus cereus*	Better physiological performance, nutrient efficiency, and increased plant growth	*Cocos nucifera* L.	Pará	[26]
*Burkholderia* sp. *Bacillus* sp. *Pseudomonas fluorescens*	Increase growth and nutrition	*Euterpe oleracea* Mart.	Pará	[27]
*Pseudomonas fluorescens Burkholderia pyrrocinia*	Modifies primary metabolism, and reduces the shadow adverse effects	*Brachiaria brizantha* syn. *Urochloa brizantha* (Hochst. ex A.Rich.) R.D.Webster	Pará	[28]
*Trichoderma Asperellum*	Biological control and photosynthetic enhancement	*Oryza sativa* L.	Pará	[29]
*B. pyrrocinia P. fluorescens*	Biological control and increased silicon efficiency	*Oryza sativa* L.	Pará	[30]
*Trichoderma asperellum*	Biocontrol and increased productivity	*Oryza sativa* L.	Pará	[31]
*Pseudomonas* sp. *Bacillus* sp. *Trichoderma* sp.	Biological control and increase the antioxidant enzyme activities	*Cocos nucifera* L.	Pará	[32]
*Burkholderia* sp. *Bacillus* sp. *Pseudomonas fluorescens*	Mitigates the effect of drought and increases the concentration of antioxidant enzymes	*Euterpe oleracea* Mart.	Pará	[33]
*Pseudomonas fluorescens Burkholderia pyrrocinia*	Increases growth and mitigates allelochemicals adverse effects	*Oryza sativa* L.	Pará	[34]
*Bradyrhizobium* sp.	Increase nutrition	*Vigna unguiculata* L. Walp	Rondônia	[35], [36]
*Agrobacterium Kluyvera*	Biological control of sclerotium wilt and promotion of growth	*Solanum lycopersicum* L.	Roraima	[37]
*Bradyrhizobium ingae*	Increases growth, nodules, and nutrition	*Inga edulis* Mart.	Roraima	[38]
*Bacillus amyloliquefaciens*	Increased growth, the concentration of phytohormones and nutrients	*Elaeis guineenses* Jacq.	Pará	[39]
*Pseudomonas* sp.	Increases growth and biomass	*Piper tuberculatum* Jacq.	Pará	[40]

**Figure 1.**
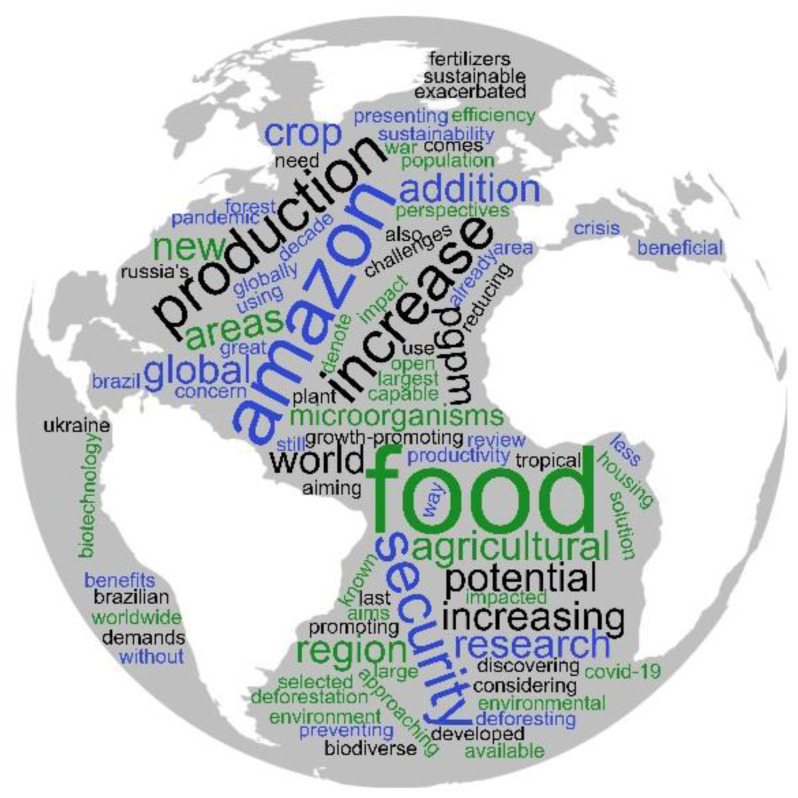
Word clouds elaborated from this study. The size of the words is correlated with the abundance of times the term was found in the selected searches.

The most frequently found terms in this search were food, Amazon, production, sustainability, safety, potential, PGPM, and microorganisms, among others, as can be seen in the word cloud in Figure 1. The survey showed that 90% of the included studies were carried out in nurseries and greenhouses, though each of these stated an explicit interest in continuing research in agronomic field trials and at larger biotechnological scales. Among the 10% of studies carried out in agricultural fields, notable successes were reported. For instance, soybeans co-inoculated with mycorrhiza and rhizobacteria in a greenhouse and in a field were studied for the potential to either increase the efficiency of phosphate fertilization or to serve as a supplement. Co-inoculation promoted a 14% increase in soybean production (5,013 kg ha-1 compared to standard inoculation 4,379 kg ha-1). The gross profit, net income, and profitability indeces of plants co-inoculated with *Bacillus* strains and arbuscular mycorrhiza were higher than standard inoculation, and co-inoculated plants had an 18% increase in the gross profit [16].

According to the survey in the Amazon region, the main benefits of microorganism-plant interactions were an increased plant growth, a greater nutritional efficiency, modifications of anatomical and morphological structures, improvements in germination, improved physiological performance and plant productivity, and a greater tolerance to biotic and abiotic stresses. Another critical application of PGPMs is their use as a biocontrol agent. For instance, *Ramichloridium* sp. was selected in Acre state in the southwestern Brazilian Amazon, to reduce anthracnose in açaí (*Euterpe oleracea* Mart.) [17]; moreover, *Colletotrichum siamense* reduced leaf lesions and increased the enzymatic activities of peroxidase, chitinase, and phenylalanine ammonia-lyase, inducing a resistance against anthracnose in guarana (*Paullinia cupana* Kunth.) [18]. These findings highlight the potential of Amazonian microorganisms to reduce the dependency on chemical pesticides and sustainably mitigate plant diseases.

PGPM-plant interactions are particularly valuable in the context of the Amazon, where the soil fertility and environmental conditions can pose challenges for agricultural and forestry systems. For example, *Trichoderma* spp. isolated from the Amazonas state demonstrated the ability to solubilize phosphate, thus resulting in over a 40% increase in the soybean (*Glycine max* (L.) Merr.) yield and more than a 100% improvement in the phosphorus absorption efficiency [19]. Such findings underscore the potential of PGPMs as biofertilizers to promote sustainable agricultural practices.

The versatility of Amazonian PGPM extends beyond agriculture. In forestry systems, arbuscular mycorrhizal fungi (AMF) have been used to restore degraded areas, such as those planted with babassu palms (*Attalea speciosa* Mart. ex Spreng.), by improving the nutrient uptake and promoting ecological success [14]. Similarly, *Burkholderia ambifaria* strains have been shown to enhance the carbohydrate and proline accumulation in guarana seedlings, thus improving their resilience under stress conditions [20]. Additionally, *Bradyrhizobium ingae* improved inga (*Inga edulis* Mart.) growth by solubilizing phosphate and AIA production, increasing the growth, biomass, root development, nodulation, and increasing the nitrogen concentration [38]. These examples highlight the multifaceted roles of PGPMs in agricultural and ecological restoration.

**Figure 2.**
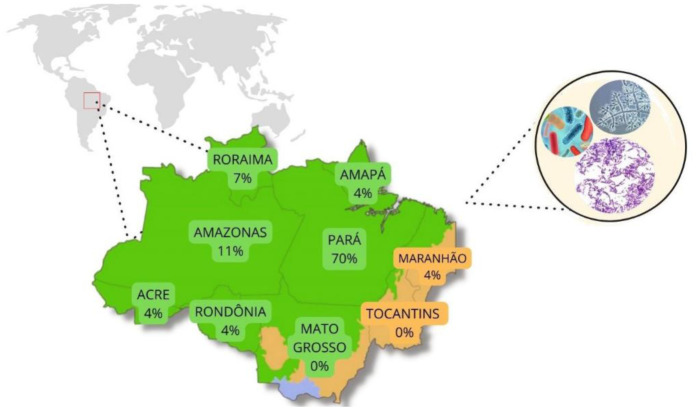
Percentage distribution of studies with plant growth-promoting microorganisms (PGPM), carried out in the last decade in the Brazilian Amazon states.

Research on this topic is increasing in the Amazonian region, especially in the state of Pará, which is in the southeastern Brazilian Amazon (Figure 2). Notable successes were reported: *Burkholderia pyrrocinia* significantly enhanced the biomass and nutrient content of emerald grass (*Zoysia japonica* Steud.) [21], while its co-inoculation with *Burkholderia pyrrocinia* and *Pseudomonas fluorescens* improved photosynthesis, nitrogen uptake, chlorophyll content, leaf area, number of tillers, and biomass production in pasture grasses (*Brachiaria brizantha* (Hochst. ex A.Rich.) Stapf. syn. *Urochloa brizantha* (Hochst. ex A.Rich.) R.D.Webster) [22]. Moreover, studies conducted in the Brazilian Amazon highlighted improved plant development in pasture areas. According to Lopes et al., (2021), biostimulants increase the production and shade tolerance in the *Brachiaria* species, thus indicating their potential as a biofertilizer in conventional and crop-livestock-forest systems [28].

Additionally, beneficial fungi and bacteria from the Amazon can protect plants against biotic stress factors. *Trichoderma asperellum* reduced rice scalds by more than 60% [29],[30], reduced the incidence of sheath blight in rice by 19%, and increased the grain yield by over 40% [31]. *Trichoderma* and rhizobacteria also act as bionematicides against *Bursaphelenchus cocophilus* (COBB) Baujard, which is the etiologic agent of red ring disease in coconut trees [32].

PGPM inoculation can attenuate abiotic stresses for the plant. Rhizobacteria inoculation in açaí seedlings mitigated the effects of drought by maintaining the photosynthetic performance and activating antioxidant enzymes [33]. In upland rice, *P. fluorescens* and *B. pyrrocinia* increase the plant tolerance to allelochemical stress, thus indicating possible practical agricultural applications to mitigate the effects of environmental allelochemistry [34]. These capabilities are particularly important for the Amazonian region, where climate change and land use pressures are increasing the prevalence of abiotic stressors.

Moreover, the research emphasizes the importance of regional biodiversity in the Amazon as a source of PGPMs. Microorganisms such as *Bradyrhizobium* sp. and *Agrobacterium* sp., isolated from the states of Rondônia and Roraima, respectively, have shown significant benefits for cowpea (*Vigna unguiculata* L. Walp.) and tomato (*Solanum lycopersicum* L.) crops by improving the nodulation, nutrient uptake, and resistance to biotic stressors such as sclerotium wilt [35],[37]. These findings underscore the unique potential of Amazonian microorganisms to contribute to sustainable agriculture while preserving the biodiversity of the Amazonian region.

Economically, PGPMs offer a cost-effective alternative to chemical fertilizers. For example, co-inoculation of arbuscular mycorrhiza and rhizobacteria in soybeans improved the profitability of the system [16]. *Bacillus amyloliquefaciens* induced precocity, and in oil palm seedlings [26], reduced the chemical fertilizer use by 50%, and lowered the production costs while mitigating environmental impacts [39], thusdemonstrating the economic viability of microbial biotechnologies.

These studies carried out in the Brazilian Amazon highlight the potential beneficial effects for different plant species of microorganisms native to this region. This biotechnology has been shown to improve the plant development and production, reduce pathologies, and mitigate environmental stresses in agricultural, forage, and forestry crops. However, further field trials and large-scale applications are needed to fully realize their potential and integrate these technologies into broader agricultural and forestry systems.

Microbial technology has been used as an alternative method to promote plant growth and induce precocity to obtain seedlings with a cost reduction, with a consequent reduction of the environmental impacts as compared to the use of chemical fertilizer. This demonstrates that this field of study has a great potential for the development of agriculture in the Amazon and represents a path that will lead to the conservation of biodiversity in the region, thereby encouraging sustainability, reducing negative environmental impacts, and increasing production to ensure food security.

## 4. Plant Growth-Promoting Microorganisms (PGPM) mechanisms

Plant-microbe interactions are benefited by microbial biotechnology in an agricultural context by reducing the incidence of diseases, promoting growth, and increasing plant production, particularly in regions such as the Amazon, where the biodiversity of microorganisms provides an invaluable resource for innovation. Microbial biotechnology has the potential to reduce the use of chemical products, soil and water contamination, enhance the soil fertility, and mitigate environmental impacts [41].

**Figure 3.**
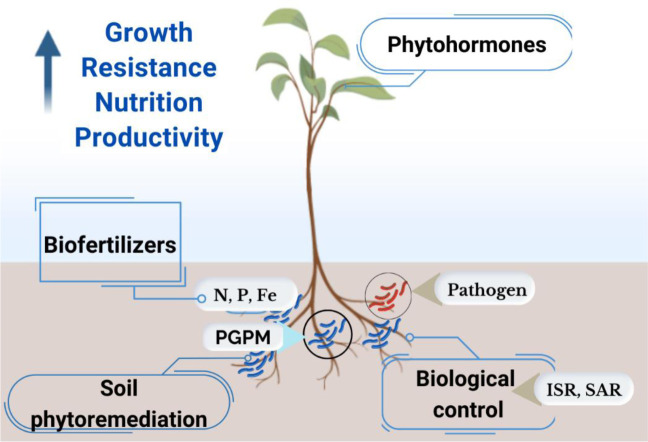
Mechanisms of action of plant growth-promoting microorganisms (PGPM) to promote growth, resistance, nutrition, and crop productivity. PGPM increases nitrogen (N) fixation, phosphorus (P) solubilization, production of siderophores (Fe), and synthesis of phytohormones (e.g., cytokinins, gibberellin, and indole-3-acetic acid - IAA). PGPM also acts in soil phytoremediation and biological control by increasing systemic induction resistance (ISR) and systemic acquired induction (SAR).

The Amazonian region, renowned for its unparalleled microbial biodiversity, has been the focus of numerous studies that explored the potential of plant growth-promoting microorganisms (PGPM). Genera such as *Bacillus*, *Pseudomonas*, and *Trichoderma* have been extensively studied for their ability to enhance plant growth and resilience. These microorganisms exhibit remarkable multifunctionality, thus contributing to plant development through biological control, nutrient solubilization, and soil phytoremediation. Additionally, they enhance the plant nutritional status, produce vital metabolites, and synthesize key phytohormones. Furthermore, PGPM are instrumental in mitigating the adverse effects of biotic and abiotic stresses, including salinity, drought, water saturation, heavy metal toxicity, temperature extremes, and damage from diseases, insects, nematodes, and viruses (Figure 3) [7],[8],[42]. This multifunctional nature positions Amazonian PGPMs as essential tools for sustainable agriculture and environmental resilience.

Beneficial microbes can be endophytic, thus inhabiting different plant tissues such as leaves stems, seeds, roots, and flowers. There are also rhizospheric microorganisms, which are associated with the root system of plants [8]. PGPM has a biochemical plasticity and can adapt to different conditions. These microorganisms play an important role in agriculture, thus improving the efficiency of chemical the inputs, controlling pathogens, and increasing the agricultural production [7],[43]. PGPM directly promotes plant development as a biofertilizer by promoting biological nitrogen fixation, phosphate solubilization, and the production of siderophores [41]. In the Amazon, species such as *Burkholderia pyrrocinia* and *Pseudomonas fluorescens* have demonstrated their ability to enhance nutrient uptake and photosynthetic efficiency in pasture grasses and upland rice, thereby supporting sustainable livestock and crop systems (Table 1).

In addition, they modulate the synthesis of plant hormones such as auxin, gibberellin, cytokinin, abscisic acid, salicylic acid, and jasmonic acid, thus increasing the synthesis of a 1-aminocyclopropane-1-carboxylase deaminase, which reduces the levels of 1-aminocyclopropane-1-carboxylate (ACC), an ethylene precursor [44]. For example, *Burkholderia ambifaria* enhanced the carbohydrate and proline accumulation in guarana seedlings, thus improving their resilience to abiotic stresses [20]. In açaí seedlings, rhizobacterial inoculation maintained the photosynthetic performance under drought conditions by activating antioxidant enzymes and stress defense pathways [33]. These capabilities position Amazonian PGPMs as critical components of integrated crop management systems.

Thus, microorganisms are also potent ecological sources of bioactive compounds, which are safer products and sustainable alternatives for managing plant diseases [6]. The mechanism with which PGPM acts as a biological control agent is indirect and involves the production of chemical compounds and enzymes that inhibit pathogen growth by degrading its cell walls, thereby reducing the competition for space and nutrients, antibiosis, and enzyme production (e.g., chitinases and glucanases) [45].

Biological control is another key mechanism of PGPMs, which involvest the production of bioactive compounds and enzymes such as chitinases, glucanases, and proteases. These compounds degrade the cell walls of pathogens, inhibit their growth, and reduce the competition for space and nutrients. For example, *Ramichloridium* sp. and *Trichoderma asperellum* stimulate systemic resistance in plants through induced systemic resistance (ISR) and systemic acquired resistance (SAR), thereby activating defense pathways that increase the production of phenolic compounds and antioxidant enzymes in açaí and rice, respectively [16],[31].

The use of beneficial microorganisms induces disease resistance, increases nutrition, and improves the plant tolerance to various stresses [46],[47]. According to Lopes et al. (2021), PGPM can change the host plant's morphology, anatomy, biochemistry, and physiology [7]. This results in an enhanced chlorophyll content, gas exchange, leaf area, root development, height, stem diameter, biomass production, seed germination, seedling vigor, nutrition, and crop productivity.

PGPMs play a role in mitigating abiotic stresses such as salinity, drought, heavy metal contamination, and allelochemical toxicity. In upland rice, co-inoculation with *Pseudomonas fluorescens* and *Burkholderia pyrrocinia* improved the root architecture and enhanced the tolerance to allelochemical stress [13],[34]. These mechanisms are important in the Amazonian region, where agricultural expansion often occurs in areas with degraded soils and challenging environmental conditions. Amazonian PGPM represents a transformative opportunity for sustainable agriculture, forestry, and ecological restoration. By enhancing the plant growth, mitigating environmental stresses, and reducing the dependency on chemical inputs, these microorganisms align with global efforts to promote sustainability and food security.

## 5. Microbial biotechnology in the Brazilian Amazon: Challenges and prospects

Microbial biotechnology employed as a biological inoculant is a sustainable technique that is easy to apply, low cost, and non-polluting, which reduces the negative environmental effects of agrochemicals, in addition to providing a greater profitability for the producer [48],[49]. In the last 10–15 years, formulated plant-beneficial microorganisms have gained a widespread acceptance as a viable alternative to agrochemical products [49]. The benefits of using PGPM have been globally researched, with 70% of research carried out in Asia, followed by America (18%) and Europe (5%), with India and China having the highest number of research studies conducted with PGPM [50].

This biotechnology has been used worldwide, and in 2021, the global revenue from the sale of these products, including bioinsecticides, biofungicides, bionematicides and inoculants, was US$ 10.6 billion. In Brazil, there has been an exponential growth of PGPM used as inoculants in the last decade, generating savings of R$ 165 million per year for the Brazilian agricultural sector, mainly because Brazil manufactures 97% of its biological inputs, which reduces spending on imported chemical inputs [51]. In the last decade, the sale of inoculants increased more than five times; for example, in 2012, 22.2 million doses were sold, in 2019 there were 70 million, while in 2022, sales rose to 134.9 million doses, with profitability in Brazil in 2023 around R$ 442 million [51],[52].

In 2022, inoculants formulated with *Bradyrhizobium*, *Azospirillum*, and *Pseudomonas* were the best-selling in Brazil, with 70% used in soybean cultivation, followed by grasses and other crops [52]. The southern and southeastern regions of Brazil are the largest consumers of inoculants, where they are used by more than 80% of soybean farmers, mainly focusing on biological nitrogen fixation (BNF), which results in savings for the farmer and benefits to the environment [53]. This represents a great opportunity to further expand research and the development of inoculants for other crops [52], including in the Amazonian region.

Furthermore, in the Amazonian region, the use of inoculants that produce phosphatases would help producers reduce the costs associated with phosphorus fertilizers, since a large proportion of phosphorus in Amazonian soils is not available to plants, thus requiring larger quantities and frequency of application of these fertilizers [54]. The use of inoculants with microorganisms capable of transforming inorganic phosphorus into dihydrogen phosphate (H_2_PO_4_^-^) or metaphosphoric acid (HPO_2_^-^) would make this nutrient available for absorption by plants [7]. In this context, the use of this PGPM would be a sustainable alternative because the fertilizer application would be reduced while the soil phosphorus retention would be increased, which would stimulate agricultural intensification in tropical environments and contribute to the global food supplies.

Research developed in the last decade highlights the potential of microorganisms native to the Amazon as a biotechnological alternative to improve agricultural production, reduce pathologies, and mitigate the environmental impacts. In this way, this technology works to conserve the region's biodiversity, promote sustainability, and increase production while also contributing to food security. However, more studies are needed to better understand how the soil microbiome can be used for the development of sustainable agriculture and restoration programs in the Amazon region. In addition, there is a need to clarify its mechanisms in plant development to aid in the process of developing potential inoculants and biocontrol agents [12],[55].

**Figure 4.**
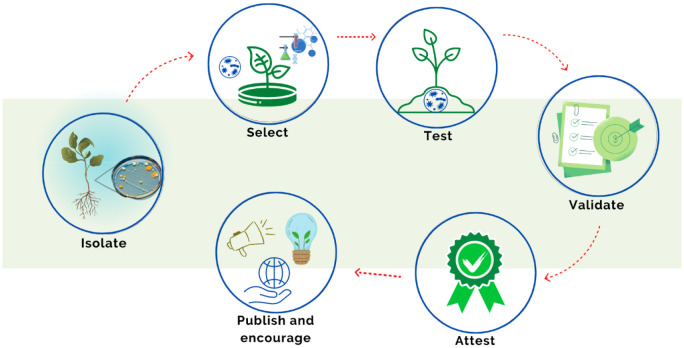
Roadmap for finding, validating, and encouraging PGPM-based products.

The isolation of PGPM, selection, testing, dissemination, and the promotion of biotechnological processes is a growing area of research worldwide (Figure 4). To increase the use of inoculants, it is necessary to combine the areas of research, production, and regulation. Universities and research institutes are essential to developing new studies to select better microorganism strains and more appropriate inoculation modes to promote efficient inoculant interactions with specific cultures, which would serve as a basis to generate new products. In this scenario, companies would play the role of developing formulations to supply production at a commercial scale, in addition to marketing the product. Furthermore, to maintain the rigor of the product quality standard, preserving the concentration, purity, and viability of the inoculant, and legalization and supervision by the Ministry of Agriculture, Livestock, and Food Supply (MAPA) are necessary [53].

The applicability of this research will only be achieved with interactions between academic institutions and industry, and a subsequent dissemination of the use of inoculants among farmers. Using the rich biodiversity of the Amazonian region as a sustainable strategy is a challenge because there is little information on the microorganism species and their potential to improve the agricultural productivity [40]. Another issue is the reduced number of institutions and researchers in the Amazon in relation to the vastness of this region.

The increasing interest in microorganism biotechnology will require additional research assistance to improve laboratories through the acquisition of modern technological equipment, in addition to increasing the microorganism selection and isolation research and testing different and more viable culture media for the growth of microorganisms. Additionally, studies must be conducted on the field survival, multiplication, longevity, strain improvement, authentication, and quantification of commercial production [8],[41]. Further research is also needed on the interference in native microbiota, different biotic stresses, and adaptation of strains in different edaphic and climatic conditions, in addition to experiments examining the inoculation of microorganisms in different cultures and geographic regions to test their survival and applicability.

Microbial technology needs to be extensively researched to obtain the desired results and acquire the trust of farmers [56]. Moreover, with regard to the Brazilian Amazon region, it is expected that, in the next decade, the amount of research to discover new microbial inoculants through field bioassays with different crops and under different biotic and abiotic stresses will increase.

Despite the evidence that this is an ecologically and economically viable biotechnology, it is still unknown to most farmers, especially small and medium-sized ones. Moreover, this makes it necessary for research companies to either adapt or associate with other specialists in disseminating knowledge to farmers and consumers about the advantages of using bioproducts. Government policies must be created to encourage the use of PGPM-based products (e.g., biofertilizers, bio-fungicides, and biopesticides). Additionally, it is necessary to encourage the management of phytopathogens and pests with a reduced use of synthetic fungicides, making bioproducts available at affordable prices, and even reducing taxes on factories that produce them and farmers who use these products [46]. It is important to have greater investments in the bioproducts industry and in new inoculation technologies, such as nanocapsules, nanoemulsions, and nanosuspensions [56].

## 6. Conclusions

The potential to optimize plant development through the biodiversity of the Brazilian Amazon microbiota, addressed throughout this study, highlights the relevance of PGPM in this region. Over the past decade, microorganisms in the Amazon biome have increased the plant growth, the nutrient use efficiency, and the resistance to disease and environmental stresses. Bacteria and fungi native to the Amazon have been shown to enhance the development of different agricultural, forestry, palm, and forage crops.

This research revealed a promising approach to develop sustainable agricultural practices based on the use of microorganisms native to the Amazon. Beneficial findings with Amazonia PGPM highlighted the need for this topic to be better explored while recognizing the challenges and perspectives it faces. Therefore, the dissemination of this research is crucial for the interaction between academic institutions, industry, and farmers. This will expand partnerships, financing, and awareness and stimulate interest in this area of study. Furthermore, issues such as research dissemination, farmer training, large-scale applicability, environmental safety, and implementation across different crops are areas that demand a continued attention.

Therefore, the potential of Amazonian PGPM to be used in the inoculant industry would benefit the agricultural production of different crops, in addition to reducing costs and the use of chemical inputs, minimizing adverse impacts on the environment, and contributing to the conservation of the Amazon. This would benefit the future of agriculture in the Amazon, thus contributing to the balance between agricultural production and environmental conservation in the region. Furthermore, the advancement of knowledge in this area is promising, which has resulted in the development of new microbial strains for the formulation of new inoculants that can increase the productivity of different crops, thus favoring global food security and the sustainability of agricultural production.

## Use of AI tools declaration

The authors declare they have not used Artificial Intelligence (AI) tools in the creation of this article.
